# A data sheet for the simultaneous assessment of dual radioactive tracer uptake in the heart

**DOI:** 10.1016/j.mex.2016.03.015

**Published:** 2016-04-02

**Authors:** Takao Kato

**Affiliations:** Department of Cardiovascular Medicine, Graduate School of Medicine, Kyoto University, 54 Shogoin Kawahara-cho, Sakyo-ku, Kyoto 606-8507, Japan

**Keywords:** A data sheet for a dual radioactive tracer method, Myocardium, Radiotracer, Data sheet, Dual tracer method, Uptake, Small animal studies

## Abstract

The myocardium takes up two major substrates: glucose and fatty acids, and various methods have been used to evaluate this uptake. Despite extensive study of radiotracer uptake-based methods, however, an easily applicable datasheet has not previously been provided. In this manuscript, an example of a method involving an easily modified data sheet based on dual tracer methods is presented.

This method, with its data sheet:

• Is applicable to all radiotracers, regardless of decay time
• Is useful, simple, and modifiable; and
• Is applicable to small animal studies.

## Graphical abstract

**Figure:**
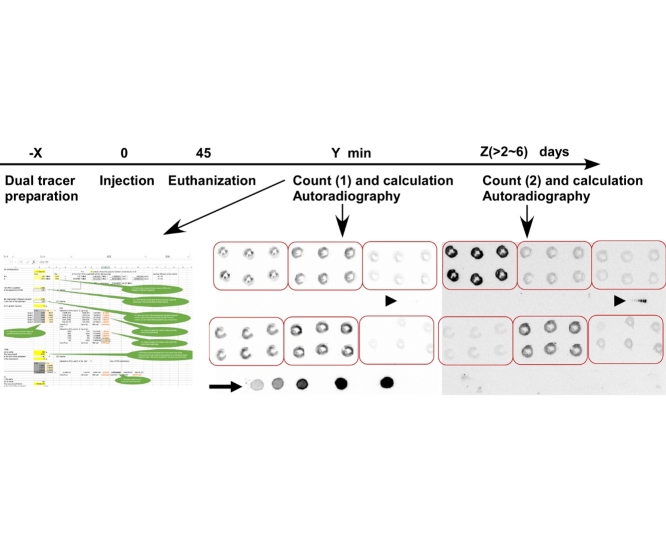

## Methods details

### Overview

**Fig. 1:**
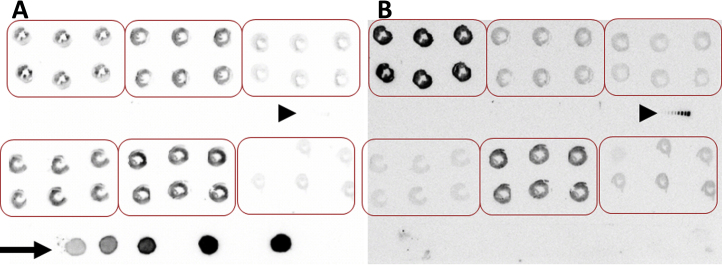
(A) Autoradiography of ^18^F-fluorodeoxyglucose (^18^FDG) at the first analysis. (B) Autoradiography of ^125^I-iodophenyl 9-methylpentadecanoic acid (^125^I-9MPA) at the second analysis. When ^18^FDG images were acquired (A), graded standards of both ^18^FDG which consisted ^18^FDG only and ^125^I-9MPA which consisted ^125^I-9MPA olny as described in second method in the section 2 in Measurement of radioisotope activity were placed on the sheet. When ^125^I-9MPA images were acquired 9 days after the first experiment (B), the same sheet was exposed; note that the ^18^FDG standards had completely decayed. The respective ^18^FDG and ^125^I-9MPA doses per rat were 436 μCi (16.1 MBq) and 11.6 μCi (0.43 MBq). The arrow indicates graded ^18^FDG standards. Arrowheads indicate graded ^125^I-9MPA standards.

In this methodological study, myocardial glucose and fatty acid uptake were measured using ^18^F-fluorodeoxyglucose (^18^FDG) and ^125^I-labeled fatty acid tracer, respectively [1], [2]. The fatty acid tracers comprise ^125^I-iodophenyl 9-methylpentadecanoic acid (^125^I-9MPA) which is incorporated and rapidly metabolized to iodophenyl-3-methylnonanoic acid by beta-oxidation and retained in the myocardium [3] and ^125^I-BMIPP, 15-(*p*-iodophenyl)-3-*R*,S-methylpentadecanoic acid, which is another fatty acid tracer and metabolized by β oxidation and retained in the myocardium [4]. The radioactive half-lives of ^18^FDG and ^125^I-labeled fatty acid tracer are 110 min and 60 days, respectively. The dual tracer methods utilize the difference in decay times, the energy peaks, and the doses injected of two tracers. For example, rats were fasted overnight, simultaneously injected with about 0.5–1 mCi of ^18^FDG and about 10–20 μCi of ^125^I-lrabeled fatty acid tracer, and euthanized by decapitation 45 min after injection. Hearts were removed from the rats and washed in cold saline. A portion comprising one third of the apical side was frozen in liquid nitrogen, and radioisotopic activity was measured using a scintillation counter. Mid-ventricle specimens were embedded in methylcellulose, cut into serial 20-μm-thick transverse sections, and analyzed using a computer-assisted imaging-processing system. The analyzed portion of the heart is provided as an analytical example. To measure ^125^I-labeled fatty acid tracer uptake, radioisotopic activity was measured at least 48 h after the initial measurements. The amount of incorporated radioisotope was reported as a percentage of the administered dose, corrected by heart weight in grams, or as a standard uptake value (SUV) of the tissue concentration (μCi/g)/injected dose (μCi)/body weight (g). Cross-talk between the two tracers was negligible (Fig. 1A and B).

## Radioisotope preparation

1. Determine the total radioisotopic activity of ^18^FDG and ^125^I-labeled fatty acid tracer
2. Mix ^18^FDG and ^125^I-labeled fatty acid tracer with saline to a final injection volume (100–200 μl × number of rats to inject plus 5) containing 500 μCi–1 mCi of ^18^FDG and 10–20 μCi of ^125^I-labeled fatty acid tracer. Note that the prepared volume should be greater than the precise volume [100–200 μl × (number of rats plus 5)]. If mice are used, an injection volume of 20–50 μl per mouse is appropriate [5], [6].
3. Fill in the date, measured dose, measured time, and numbers used for preparation, as well as the time at the start of the experiment in the data sheet (supplemental materials). This will allow automatic calculation of the radioisotope activity at the start of the experiment.

### Animal methods

Animal care and experimental procedures were approved by the Institutional Animal Care and Use Committee of Kyoto University and conducted following the Guide for Care and Use of Laboratory Animals published by the United States National Institutes of Health.

1. Assess the body weight and cardiac function in advance. Monitor blood glucose using a self-monitoring blood glucose kit to determine whether ^18^FDG uptake is influenced by blood glucose levels.
2. In rats fasted overnight, insert a plastic needle (24-gauge) attached to a thin tube containing saline into the tail vein. Inject a 100–200-μl volume containing 1 mCi of ^18^FDG and 20 μCi of ^125^I-labeled fatty acid tracer simultaneously, followed by saline to wash the tube.
3. Euthanize animals via decapitation or deep sedation 45 min after injection; remove the hearts and place them in cold saline.
4. Collect blood, wash hearts quickly in saline, and weigh each heart
5. Embed mid-ventricle specimens in methylcellulose, and freeze them on dry ice. Collect and weigh a one-third portion of the apical side and freeze this tissue in liquid nitrogen. This will be used to measure radioisotopic activity with a scintillation counter. The analyzed portion of the heart is provided as an analytical example.

### Measurement of radioisotope activity using a scintillation counter

1 Count the levels of ^18^FDG or ^125^I-labeled fatty acid tracer in the above-described apical portion via direct measurement with a scintillation counter. Window levels should be set in advance at 15–75 KeV for ^125^I and 250–750 KeV for ^18^F. The energy peak of ^18^FDG is 511 KeV; that of ^125^I-9MPA is 35 KeV. The counting efficiency specific to the scintillation counter should also be known before the experiment. For this manuscript, a 12-s counting period was appropriate for the proscribed settings and doses. The first count is acquired to determine the ^18^F uptake in the one-third apical portion of the heart. This data should be filled in the yellow-colored cells of the second Excel sheet. In the first experiment, the ^18^FDG activity was not influenced by the ^125^I-labeled fatty acid tracer activity due to the narrow energy peak and difference in the injected dose; however, the ^125^I-labeled fatty acid tracer activity measured during the first analysis exceeded the actual values; ^18^FDG has a relatively scattered energy peak and these values are not usually used in the analyses. Fill in the values in the second Excel sheet attached as a supplemental Excel file.
2 Two methods exist for the preparation of graded standards
  - The first involves a count of the mixed radioisotopes for injection. Prepare duplicates of the following: 5–10 μl of the mixed radioisotopes (A), 5–10 μl of a 2× volume of the mixed radioisotopes diluted in saline (B), 5–10 μl of a 4× volume of the mixed radioisotopes diluted in saline (C), 5–10 μl of an 8× volume of the mixed radioisotopes diluted in saline (D), and 5–10 μl of a 16× volume of the mixed radioisotopes diluted in saline (E). These will be used to calculate standard curves of known injected doses and actual counts. Count, seal, and preserve these standard samples. Note: do not discard these standard tubes because they will be needed for the second count and ^125^I-labeled fatty acid tracer calculation. Fill in the time of graded standard measurement, the volumes of graded standards (usually the same volume), and radioisotope activity in the first Excel datasheet (Supplemental materials)
  - The second method is the count of each of the used radioisotopes respectively, for which two separate graded standards will be prepared. Prepare duplicates of the following: 5–10 μl of the prepared ^18^FDG (pre-mixed) (A), 5–10 μl of a 2× volume of the prepared ^18^FDG (pre-mixed) diluted in saline (B), 5–10 μl of a 4× volume of the prepared ^18^FDG (pre-mixed) diluted in saline (C), 5–10 μl of an 8× volume of the prepared ^18^FDG (pre-mixed) diluted in saline (D), and 5–10 μl of a 16× volume of the prepared ^18^FDG (pre-mixed) diluted in saline (E). Again, the same procedure is performed for ^125^I-labeled fatty acid tracer. Prepare duplicates of the following: 5–10 μl of the prepared ^125^I-labeled fatty acid tracer (pre-mixed) (A), 5–10 μl of a 2× volume of the prepared ^125^I-labeled fatty acid tracer (pre-mixed) diluted in saline (B), 5–10 μl of a 4× volume of the prepared ^125^I-labeled fatty acid tracer (pre-mixed) diluted in saline (C), 5–10 μl of an 8× volume of the prepared ^125^I-labeled fatty acid tracer (pre-mixed) diluted in saline (D), and 5–10 μl of a 16× volume of the prepared ^125^I-labeled fatty acid tracer (pre-mixed) diluted in saline (E).
3 Count the actual administered radioisotope activities. Note that 5 μl of the injected isotope mixture (triplicate) should be counted. Fill in the time of measurement and radioisotope activity on the first Excel sheet (see a Supplemental Excel file). Note: do not discard these tubes because they will be needed for the second count.
4 After 2 or more days, perform a second ^125^I count (half-life = 60 days) of both the sample and standard tubes. Fill in the yellow-colored cells on the second Excel sheet (see also a Supplemental Excel file). The activity of ^18^FDG during the second analysis is almost identical to the background activity; hence, the count of ^125^I activity was not influenced by ^18^FDG activity.

## Autoradiography

1. Prepare the ^18^F- or ^125^I-labeled graded standards. First, prepare the measured radioisotope. For example, to evaluate ^18^FDG, 5 μl of samples (A)–(E) are placed on filter paper. The spotted filter paper is then sealed in a thin plastic wrap and placed on a sheet sized to fit the imaging plate or X-ray film (arrow in Fig. 1A). In Fig. 1A, graded standards that contained only ^18^FDG were used following preparation as described above (second method) in the section 2 in Measurement of radioisotope activity using a scintillation counter. For ^125^I-labeled graded standards, previously used graded standards (for example, an arrowhead in Fig. 1A and 1B) are prepared and placed on the sheet to account for the longer half-life.
2. Place serial 20-μm-thick transverse sections of methylcellulose-embedded mid-ventricle specimens on a prepared slide with a glass coverslip (6 slices per heart). These prepared slides should be attached to a paper sized to fit the imaging plate along with the graded standards.
3. After preparing the paper sheet with attached slides and graded standards, place the sheet upside-down on the imaging plate such that the covered glass or plastic membrane touches the imaging plate or X-ray film directly for 1 h. Next, analyze the imaging plate using a computer-assisted imaging-processing system (BAS 1000, Fuji Photo Film Co., Ltd., Tokyo, Japan) to acquire photo-stimulated luminescence or X-ray film to acquire an image. If using the BAS system, a linear correlation should be fitted between the relative activities (Photo-Stimulated Luminescence; PSLs) of the graded scales and actual radioisotopic activities (see detailed in explanation of the data sheet and a Supplemental material). If using X-ray film, densitometry should be performed, and the results should be fitted between the densities of the graded standards and actual radioisotopic activities. The dose and energy of ^18^FDG is enough large that the activity of the ^125^I-labeled fatty acid tracer might be negligible in the first exposure (arrowhead in Fig. 1A).
4. After obtaining the ^18^FDG image (Fig. 1A), the imaging plate should be removed, and the next exposure prepared.
5. After 2 or more days, perform the next exposure in the same way (Fig. 1B), using the above-mentioned paper sheet. As the ^18^F will have completely decayed, all radioisotopic activity will be derived solely from ^125^I. The exposure time is longer than that used for ^18^F; for example, an exposure of 3–4 days is appropriate, due to the low injected dose and weak energy peak of ^125^I.

## ^99m^Tc-sestamibi (MIBI) and ^125^I-labeled fatty acid tracer

If ^99m^Tc-MIBI is used as an indicator of mitochondrial membrane potentials, which has a half-life of 6 h, an injected dose of 12.5 MBq (337.8 μCi) per rat will not cause cross-talk with ^125^I-labeled fatty acid tracer with an injected dose of 0.37 MBq (10 μCi) [7]. The first analysis is done to acquire ^99m^Tc activity. If using the Excel sheet, change 110, the half-life of ^18^FDG, to 240, the half-life of ^99m^Tc, in the calculation formula. Because of this longer half-life, ^125^I should be measured 7 or more days after the first experiment.

**(A)** (Actual values at the start of the experiment) = (Measured values of samples) × (0.5)^(−t/240)^,

where t is the elapsed time between the start of the experiment and the measurement of the samples.

## ^201^Tl (thallium) and ^125^I–labeled fatty acid tracer

If ^201^Tl is used as a myocardial perfusion tracer, which has a half-life of 73 h, an injected dose of 160–640 μCi per rat will not cause cross-talk with ^125^I-labeled fatty acid tracer with an injected dose of 16–64 μCi per rat [8]. The first analysis is done to acquire ^201^Tl activity within 11 h. If using the Excel sheet, change 110 (min), the half-life of ^18^FDG, to 4380 (min), the half-life of ^201^Tl, in the calculation formula.

**(B)** (Actual values at the start of the experiment) = (Measured values of samples) × (0.5)^(−t/4380)^,

where t is the elapsed time between the start of the experiment and the measurement of the samples.

Because of this longer half-life, ^125^I should be measured 30 or more days after the first experiment. The exposure time for autoradiography needs 3–4 days in BAS system and a longer period in X-ray film.

## Explanation of the excel sheet

How to use this sheet attached as a Supplementary Excel file.

1. Open the first sheet. Fill in the date (cell B2), measured dose (cells B4 and B5 in units of Ci [curies] and cells B7 and B8 in units of Bq [becquerels]), measured time (cells D4 and E4, D5 and E5 or D7 and E7, D8 and E8), and number of injections prepared (numbers of animals plus 5, cell I2). Fill in the time at the start of the experiment (cell B12 and C12). Calculate the radioisotopic activity at the start of the experiment (cells G4 and G5 or G7 and G8).
  Calculation formulas are as follows:
  For ^125^I activity: Because of the long decay time, the activity at the start of the experiment is almost the same as that of the measurement (cells G4 and G7).
  For ^18^F activity:
  (C) (Actual values at the start of the experiment: cells G5 and G8) = (Measured values at the time of measurement of preparation) × (0.5)^(t/110)^,
  where t is defined as the elapsed time between the time of the start of the experiment and the measurement (cell E13), and 110 is the half-decay time of ^18^F.
  Calculate the radioisotopic counts of the injected isotope (cells N4 and N5 or N7 and N8) according to the gamma camera counting efficiency (P4 and P5). The counting efficiency differs for each camera; accordingly, each camera uses a different calculation formula. In general, however, the radioisotopic counts of the injected isotope (cells N4 and N5 or N7 and N8) should = Correcting coefficient (the counting efficiency) x (actual values; L4 and L5 or L7 and L8) (Eq. (D)).
2. Open the second sheet. Fill in the cells indicating the identification number of each animal (cell D6), animal group (cell C6), animal body weight (cell E6), date of the experiment (cell F6), the time of the start of the experiment (cells W4 and X4), and blood sugar concentration (cell G6) before injection.
3. After euthanization, record the weight of the whole heart into the appropriate cell (cell H6) in the second Excel sheet. The mid-ventricle and apical one-third portions would be excised; the latter is then weighed, and this value is filled into the cell (cell I6) in the second sheet. The weight of the collected blood is also determined and entered into the second Excel sheet (cell J6); note that the weight of the empty collection tube should be measured previously, and the weight of blood should thus be corrected.
4. Measure the radioisotope activity of the heart and blood fill in the yellow-colored Excel cells (K6 to K11 and M6 to M11, Q6 to Q11 and S6 to S11, respectively) and the time of measurement on the second Excel sheet (cells O6 and P6). If the gamma counter expresses the actual values (such as nCi or MBq) of the specimens, these values can be filled in directly in cells L6 to L11 and N6 to N11 or R6 to R11 and T6 to T11. Measure that of the blood sequentially.
5. Measure the activities of graded standards and record these values in the first Excel sheet (cells B23 to B27 and cells C23 to C27). Fill in the time of the measurement of graded standards (B17 and C17). The actual values are calculated (cells F23 to F27 and F31 to F35) according to Eq. (D). The time after the experiment is calculated (cell E8). ^18^F at the start of the experiments is calculated (cells H23 to H27 and cells J23 to J27). If the gamma counter expresses the actual values (Bq) of the specimens, fill in these values in cells F23 to F27. If the values have been adapted to Ci, take care to replace Bq with Ci in the sheet.
6. Count the actual administered radioisotopic activities. A total of 5 μl of the mixed isotopes (triplicate) should be counted. Fill in the volume injected (cell B40 in the first Excel sheet), the volume of the measurement (cell B42), the time of measurement (cells B44 and C44) and calculation (cell E45), and the radioisotopic activity (cells C49, C50, and C51) on the first Excel sheet. The average counts are calculated (C49) and then subsequently converted to the actual values (cells F49 to F52), and the values at the start of the experiment are calculated (cell H52). This value is converted to the injected value (cells M52 and N52). The ^125^I counts at the start of the experiment are also calculated. Fill in these counts into cells B23 to B27 and calculate the actual values (cells F31 to F35), or directly fill in the actual values into cells F31 to F35.
7. Copy and paste data concerning the initial radioisotope activity (actual values) injected per animal from the first Excel sheet to the second Excel sheet (automatically filled into cells Q37 and Q38 in the second Excel sheet).
8. On the second Excel sheet, calculate the activity per gram of tissue, the% dose/g, and SUV (standard uptake value).
  (E) (Actual values at the start: cell K18) = (Measured values of a sample: cell N6) *(0.5)^(−t/110)^,
  where t is the elapsed time (cells W6 or L18) between the start of the experiment and the time of the measurement of the sample.
  (Activity per gram) = (Actual values at the start of the experiment)/(tissue or blood weight [g])
  (the% dose/g) = (Activity per tissue gram)/(injected radioisotope activity)
  (SUV) = (the% dose/g) × (body weight)
9. During the second count for ^125^I activity, the ^125^I activity values should be filled in where appropriate. Count the actual radioisotope activities of 5 μl of the injected (mixed) radioisotope (triplicate) and fill in the first Excel sheet (cells B62 to B64). In addition, fill in the time of measurement (cell B57) and calculation of days form the first experiment (cell B58). The ^125^I at the start of the experiments are calculated (cell H65) according to the Eq. (F), and copy and paste these data to the second sheet (cell Q37).
  (F) (Activity at the experiment) = (measured activity) × (0.5)^(−t/60)^
  where t is defined as the days from the first experiment. 60 (days) is the half decay time of ^125^I.
10. Measure the ^125^I activities of the heart and blood, and fill in these values (cells AD6 to AD11 and AF6 to AF11, or directly into cells AE6 to AE11 and AF6 to AF11 if a gamma camera yields actual values) and the date of measurement (cell AF3) on the second Excel sheet. On the second Excel sheet, calculate the activity per gram of tissue, according to Eq. (F).
11. For both ^18^FDG and ^125^I-labeled fatty acid tracer autoradiography, acquire the PSL and calculate PSL/mm^2^. For X-ray film, acquire the image and calculate the image density instead of the PSL values. Calculate the PSL of the heart minus background PSL. This calculation is performed by fitting values to a standard curve generated using graded standards and their known radioisotope activity levels. An example of the third and the fourth Excel sheets is provided in Fig. 1A and B.

## Discussions

Recent technical advantages have enabled system-wide measurements of metabolites in various organisms [9], [10], [11]. Two tracers, one radioactive and one stable, are used in these experiments. The advantages and disadvantages of these tracers are briefly summarized in Table 1. Dual-tracer methods do not limit the use of radioactive tracers, and both tracers can be used in a dual-tracer method. In this methodological paper, a dual-tracer method based on 2 radioactive tracers is introduced.

## Conflicts of interest

No declared.

## Source of funding

The funding source had no role in this study.

## Figures and Tables

**Table 1:** Advantages and disadvantages of radioactive and stable tracers.

	Advantages	Disadvantages
Radioactive	In vivo imaging available by PET or SPECT; relatively easy clinical application Autoradiography	Some tracers have a very short decay time
	systems and activity counters widely available for ex vivo analysis	Some tracers are trapped, and only substrate uptake is analyzed
	Dual-tracer methods utilize different decay times, energy peaks, and dosages	Limited information available about intermediate metabolites

Non-radioactive (Stable tracers)	Fluxome analysis of intermediate metabolites from tracers using nuclear magnetic resonance (NMR) or gas chromatography mass spectrometry (GC–MS)	Difficulties with in vivo analysis
	Complex metabolic networks can be analyzed	Expensive tracers and equipment
	Do not decay or emit radiation	Natural abundance of a given isotope (and presence of multiple other isotopes) must be low
		Amounts should be sufficiently large to account for when calculating a metabolic rate
